# Eye movement behavior in a real-world virtual reality task reveals ADHD in children

**DOI:** 10.1038/s41598-022-24552-4

**Published:** 2022-11-24

**Authors:** Liya Merzon, Kati Pettersson, Eeva T. Aronen, Hanna Huhdanpää, Erik Seesjärvi, Linda Henriksson, W. Joseph MacInnes, Minna Mannerkoski, Emiliano Macaluso, Juha Salmi

**Affiliations:** 1 Department of Neuroscience and Biomedical Engineering (Otakaari 3), Aalto University (AALTO), P.O. BOX 00076, Espoo, Finland; 2 VTT Technical Research Centre of Finland, Espoo, Finland; 3 Child Psychiatry, University of Helsinki and Helsinki University Hospital, Helsinki, Finland; 4 Laboratory of Developmental Psychopathology, Pediatric Research Center, New Children’s Hospital, Helsinki, Finland; 5 Child Neurology, University of Helsinki and Helsinki University Hospital, Helsinki, Finland; 6 Department of Computer Science, Swansea University, Swansea, UK; 7 Lyon Neuroscience Research Center (UCBL1 INSERM-U1028, CNRS-UMR5292), Lyon, France; 8 Aalto Behavioral Laboratory (ABL), Aalto University, Espoo, Finland; 9 MAGICS, Aalto Studios, Aalto University, Espoo, Finland

**Keywords:** Attention, Diagnostic markers, Human behaviour

## Abstract

Eye movements and other rich data obtained in virtual reality (VR) environments resembling situations where symptoms are manifested could help in the objective detection of various symptoms in clinical conditions. In the present study, 37 children with attention deficit hyperactivity disorder and 36 typically developing controls (9–13 y.o) played a lifelike prospective memory game using head-mounted display with inbuilt 90 Hz eye tracker. Eye movement patterns had prominent group differences, but they were dispersed across the full performance time rather than associated with specific events or stimulus features. A support vector machine classifier trained on eye movement data showed excellent discrimination ability with 0.92 area under curve, which was significantly higher than for task performance measures or for eye movements obtained in a visual search task. We demonstrated that a naturalistic VR task combined with eye tracking allows accurate prediction of attention deficits, paving the way for precision diagnostics.

## Introduction

Human goal-directed behavior is characterized by a constant interaction between the individual and the environment. Our natural environments are open-ended, dynamically changing, and stimulus rich, posing high demands to adaptive behavior. At the same time, such complex conditions make identifying real-life attention deficits a difficult research challenge^1,2^. Attention deficit hyperactivity disorder (ADHD) is perhaps the most widely noticed condition in which the diagnostic criteria are strongly linked with challenges to manage everyday goal-directed behaviors^3,4^.

Despite substantial efforts to develop behavioral markers over the past half a century, ADHD assessment addressing inattention, impulsivity, and hyperactivity as main symptom domains, still relies on interviews, questionnaires, and clinical observations that are limited by subjective bias and context-dependence^2^. While objective and quantitative ADHD markers should be able to account for the large phenotypic heterogeneity and symptom variability^5,6^, the prevailing experimental psychology tradition has, on the contrary, heavily constrained behavioral sampling with the use of impoverished stimuli and tasks measuring narrow cognitive constructs (e.g., sustained attention, orienting of attention, distraction)^5,7^. The tasks used in traditional experimental psychology provide a straightforward interpretation, which can be reliably linked to cognitive brain functions. This approach has allowed to accumulate valuable knowledge on attention and executive function deficits. However, at least so far, such tasks have provided limited support for ADHD diagnostics^2^. Recent developments in game engines and virtual reality (VR) have provided new opportunities to complement existing knowledge by to obtaining rich behavioral data in ecologically valid settings, with limited sacrifices on the stringent experimental control^8–11^.

We have recently developed the first VR task, named EPELI (Executive Performance in Everyday LIving), that allows quantification of attention and executive function deficits in more realistic open-ended conditions resembling situations where the ADHD symptoms are manifested^12^. In a previous study, we reported performance for five task measures that successfully discriminated ADHD children from typically developing children^12^. Despite its high potential, there is a lack of VR studies examining eye movements in ADHD individuals in naturalistic conditions^11^. Such approach could enable studying visual attention at much higher precision than task performance measures. Previous studies with restricted tasks have reported group differences between ADHD children and typically developing controls in fixations and saccades, reflecting depth of the visual information processing and orienting of visual attention^13–16^. Based on these studies, ADHD manifests as deficits of goal-directed attention, such as reduced ability to suppress saccades^13,15,17^, less accurate saccades to target location, and difficulties in maintaining fixation on a target object^14^. Moreover, problems of stimulus-driven attention, which refers to engagement of attention via some external factor in the environment^18^, manifests as a bias toward salient locations in ADHD children^19,20^. Saccades in ADHD children have shorter latencies, lower peak velocities, and less accurate landing^14,21,22^. While these studies provide important insights on aberrant attention-related processes in ADHD, what remains unresolved is whether the observed effects generalize to lifelike situations. In order to identify the mechanisms that underlie ADHD symptoms in the real life, eye movement measures could be brought to more naturalistic tasks where the participants are able to move their head, freely navigate, and interact with objects^23^. However, previous ADHD studies using VR eye tracking either have not used naturalistic paradigms or have focused on the gaze and scan path analysis rather than on conducting an in-depth analysis for the fixation and saccade parameters^11,24,25^.

Besides particular subfunctions of attention, ADHD is associated with increased fluctuations of attention over time. In eye movement research, a distinction between ambient (long saccades and short fixations scattered over wider area) versus focal (shorter saccades and longer fixations within a more restricted area) mode has been made^26,27^. With static stimuli, ambient mode typically changes to focal mode over time, while during a dynamic stimulus the participants start to switch between the two modes^26,28^. At the level of task performance, the fluctuation of attention over time is one of the most promising behavioral markers of ADHD^29,30^, but it remains unclear whether this manifests to eye movement features, for example, mode switching.

Visual saliency is usually defined based on physical property of the image, and it indicates how much a particular area of a visual stimulus stands out in its color, orientation, intensity, or other low level visual characteristics^31,32^. Saliency is considered as a main factor to guide human attention in absence of an active task (“free viewing” condition)^1,31–33^. However, it may also interfere task performance^34^. Previous studies suggest that people with ADHD tend to orient their attention to highly salient stimuli easier than participants in a typically developing control group^19,35^, which can be indicated by higher rate of saccades towards salient location, and longer fixation time on that area (including higher error rate in anti-saccade task)^21,36,37^. Neuroimaging studies also indicated abnormal functioning of the salience network in the brain in people with ADHD^20,38^. Two major attentional mechanisms that guide selective attention are stimulus-driven (bottom-up, saliency-related) exogeneous shift of attention and voluntary goal-directed (top-down) attention regulation^32^. Thus, during an active task performance number, and duration of fixations on salient locations can indicate distraction^39,40^, and potentially it can be used to indicate attentional lapses of children with ADHD^11^.

In this study, we collected eye movement data while the participants performed EPELI VR task (see https://tinyurl.com/bdh7472e). EPELI includes 13 task scenarios where the participants perform everyday chores in a virtual environment. Each task scenario has a general topic (e.g., morning routines or coming back from school), and it consists of four to six subtasks (e.g., put backpack to our room, wash your hands, eat lunch). At the beginning of each task scenario, an animated dragon character gives instructions regarding all subtasks to be done. Task scenario finishes if all the subtasks are done, or if the time limit of 90 s is reached. The total duration on the game is 25–35 min and it includes 70 subtasks.

In this study, we collected eye movement data of the participants playing EPELI with the aim of further improving the detection of ADHD and clarifying the specific mechanisms of attention deficits. In a previous study^12^, we reported performance for five task measures reflecting key aspects in ADHD symptoms. Compared to controls, ADHD children showed a higher percentage of irrelevant actions out of all actions suggesting poor attentional and executive control, longer navigation paths indicating problems with planning, tendency to perform excessive number of actions possibly associated with impulsive behavior, lower number of correctly performed tasks reflecting difficulty in executing the tasks from memory, and higher amount of controller movement that was used to index motor activity level. We expected that these finding would be replicated, and that rich data provided by fixations and saccades during this task would further improve the prediction of group status. More specifically, our preregistered hypothesis (https://tinyurl.com/yck2y7u2) based on findings of studies with conventional experimental tasks was that salient objects in the environment would attract the gaze of ADHD children more effectively than in typically developing children and that focusing attention to relevant stimuli would be less efficient in ADHD children. Finally, we expected that ADHD group would demonstrate higher number of switches between ambient and focal processing due to fluctuating attention.

## Results

### Group characteristics

The groups were matching on age, and gender, but ADHD participants came from lower wealth families and performed worse in Wechsler Intelligence Scale for Children^41^ (WISC-IV; see Table 1 and Supplementary Results: Questionnaires). The groups did not differ in terms of gender balance (Fisher's Exact Test, *p* = 0.085), but the analysis adjusted by gender is provided in Supplementary Results.

**Table 1:** Background characteristics of the study sample.

Variable	ADHD group n (percentage)	Control group n (percentage)	Test statistics	*p*
Gender	29 (78%) – boys, 8 (22%) – girls	21 (58%) – boys, 15 (42%) – girls	Fisher's Exact Test	0.081
	**Mean (SD)**	**Mean (SD)**		
Age	10 Years 5 Months (1 Year)	10 Years 9 Months (1 Year 2 Months)	t(69) = 1.62	0.11
Parental Income*	3.7 (1.1)	4.6 (0.7)	t(66) = 4.34	< 0.0001***
WISC-IV Similarities	9.2 (3.4)	11.2 (2.4)	t(63) = 2.80	0.017*
WISC-IV Matrix Reasoning	8.8 (4.0)	10.9 (3.5)	t(69) = 2.37	0.030*
ADHD-RS	31.9 (8.3)	6.2 (6.5)	t(66) = 14.63	< 0.0001***
BRIEF	195.6 (25.0)	120.3 (24.1)	t(69) = 12.93	< 0.0001***
CBCL	56.4 (23.0)	16.2 (14.4)	t(59) = 8.85	< 0.0001***
EQELI	46.8 (14.8)	16.3 (12.7)	t(68) = 9.35	< 0.0001***
Familiarity of the Tasks	4.8 (1.7)	5 (1.1)	t(61) = 0.57	0.76
Object Naming Task	38.0 (1.6)	38.8 (1.4)	t(70) = 2.18	0.13
Simulator Sickness Questionnaire	0.65 (1.0)	0.67 (1.1)	t(70) = 0.073	0.94
Presence Questionnaire	70.4 (15.6)	72.3 (10.5)	t(63) = 0.59	0.76

*ADHD-RS* ADHD Rating Scale-IV, *BRIEF* the Behavior Rating Inventory for Executive, *CBCL* the Child Behavior Checklist, and *EQELI the Executive Questionnaire of Everyday LIfe* (see Methods section and Supplementary Methods: EPELI. After-game assessment).

* Average parental income before tax was reported as: 1 = *less than 1 500 €/m, 2* = *1 500–2 200 €/m, 3* = *2 200–3 000 €/m, 4* = *3 000–4 000 €/m, 5* = *over 4 000 €/m; before tax per adult.*

### EPELI results

**Figure 1:**
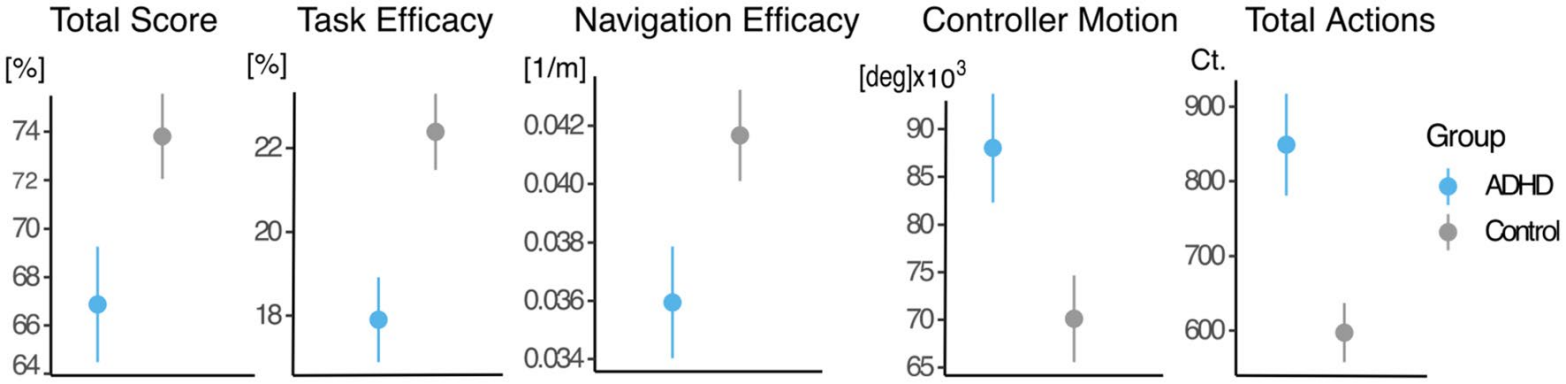
The plot shows mean of the EPELI performance measures in the ADHD and control group. Error bars indicate the standard error of mean.

Task performance measures: We first examined group differences in five measures reported by Seesjärvi and colleagues^12^ (see Fig. 1). A group difference was observed in all EPELI performance measures, with the ADHD group having lower Total Score (Z = 2.31, *p* = 0.021), Task Efficacy (Z = 3.1, *p* = 0.0048), and Navigation Efficacy (Z = 2.73, *p* = 0.0085), and higher level of Controller Motion (Z = 2.7, *p* = 0.0085) and Total Actions (Z = 3.2, *p* = 0.0048) than the control group.

To examine how well we could identify the group membership of individual participants, we trained a supported vector machine^42^ (SVM) classifier using data of the five EPELI performance measures reported above (SVM 1). This analysis resulted in 0.77 Area Under the Receiver Operator Characteristic Curve (AUC score).

#### The effects of fixations and saccades across the experiment

 A mixed linear model^43^ was applied to analyze Fixation Duration, Saccade Duration, and Saccade Amplitude. The eye movement properties were modeled as a linear function with Group and Task Scenario as fixed effects and Participant and Task Scenario Order as random effects (intercepts, see Table 2). A possible distractor effect in the eye movements (see Supplementary Results: Distractor Effect) was accommodated by including Task Scenario Order as a random factor, as the task scenarios including distractors varied with the Scenario Order.

**Table 2:** Test statistics and Mixed Effect Models for the main eye movement features.

Dependent Variable	ADHD group: Mean (SD)	Control group: Mean (SD)	Effect	χ^2^	Df	φ	p
Fixation Duration	317 (36) ms	309 (30) ms	Task Scenario	128.6	12	1.43	< 0.0001 ***
			Group	3.93	1	0.25	0.047 *
			Task Scenario × Group	25.47	12	0.64	0.038 *
Saccade Duration	57 (8.3) ms	67 (10.1) ms	Task Scenario	186.5	12	1.74	< 0.0001 ***
			Group	17.95	1	0.57	< 0.0001 ***
			Task Scenario × Group	13.20	12	0.49	0.35
Saccade Amplitude	5.44 (1.4) deg	6.29 (1.7) deg	Task Scenario	243.5	12	2.01	< 0.0001***
			Group	9.99	1	0.44	0.0023*
			Task Scenario × Group	18.94	12	0.56	0.14

**Figure 2:**
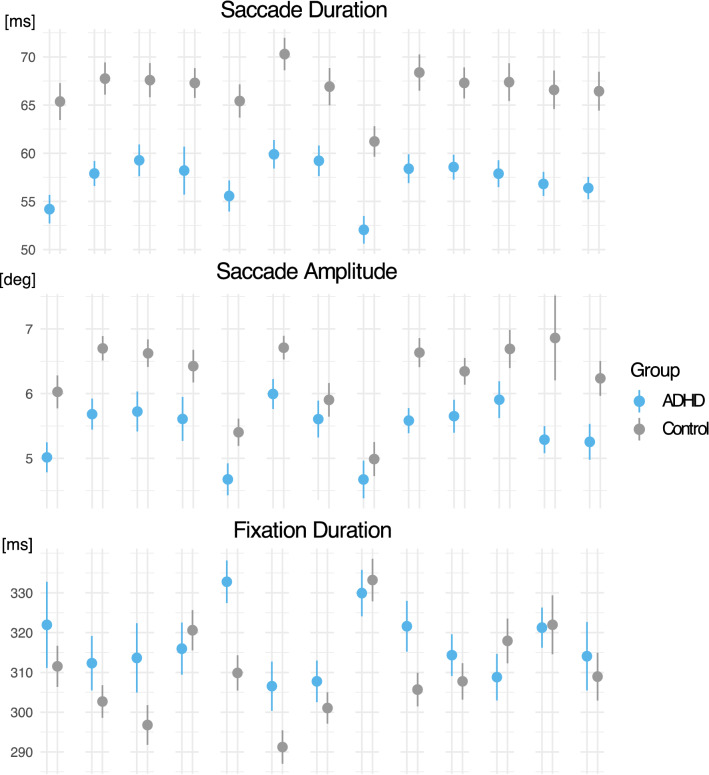
The effects of Task Scenario and Group on the eye movement features. Columns represents each EPELI task scenario. Error bars indicate the standard error of mean.

The mixed model showed that participants in the ADHD group had overall shorter Saccade Duration with smaller Saccade Amplitude, and this difference was consistent across EPELI task scenarios (see Fig. 2). Fixation Duration was longer in the ADHD group as compared with the control group, but the effect was moderated by the Task Scenario (see Table 2 and Fig. 2). Additional models which test eye movement difference between the groups adjusted by gender provided in Supplementary Materials (see Supplementary Results: Statistical Analysis Adjusted for Gender).

Correlation between the eye movement features and total score on ADHD-RS symptoms questionnaire can be found in the Supplementary Materials (Supplementary Results: Correlation between Eye Movements and ADHD-RS score).

**Figure 3:**
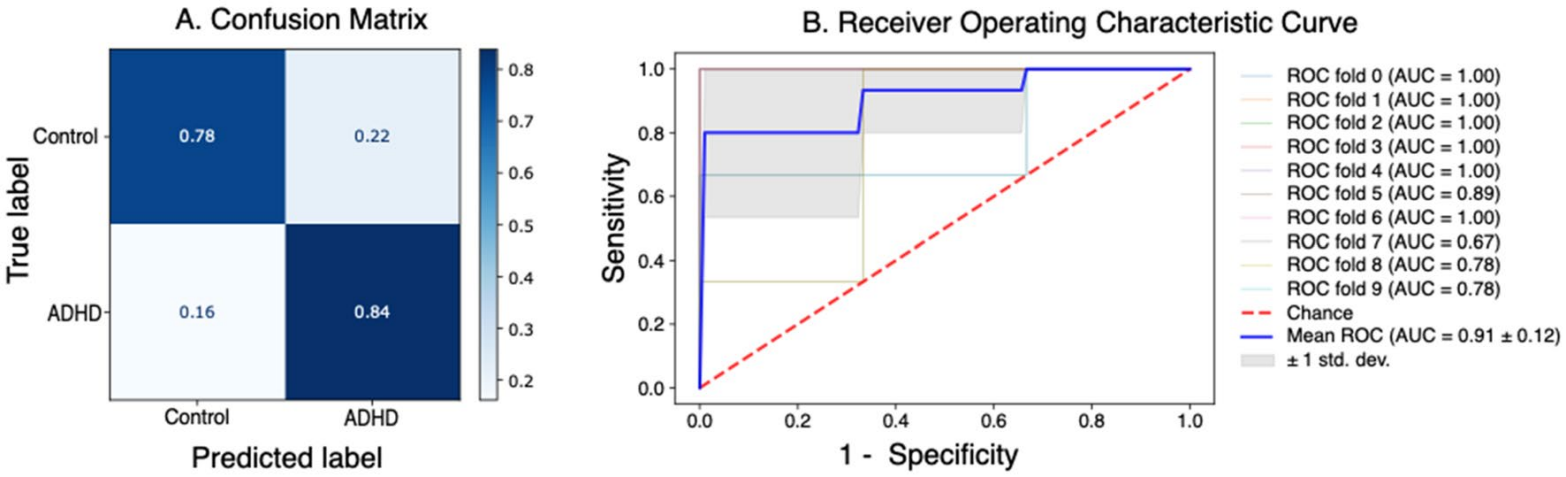
The figure shows evaluation of the SVM classifier trained on eye movement features. (**A**) Confusion matrix shows percentage of correctly classified and mislabeled participants per group averaged on tenfold cross validation, (**B**) the panel shows ROC curve and AUC scores for each cross-validation fold and the average.

To test our main research question related to the predictive ability of eye movement features in EPELI, we trained an SVM classifier (SVM 2) on Fixation Duration, Saccade Duration, and Saccade Amplitude data aggregated per task scenario. The classifier demonstrated an excellent AUC score of 0.92 after tenfold cross-validation (see Fig. 3 for the confusion matrix and the ROC-curve plot), which was higher than the performance of SVM 1 (t(58) = 8.48, *p* < 0.0001).

#### The effect of task performance on the eye movements

 To clarify the role of task performance on group differences in eye movement features, we repeated the mixed models testing the group differences by regressing out the effect of Total score. Group differences in adjusted Saccade Duration (χ^2^(1) = 14.60, φ = 0.48, *p* = 0.00040), and adjusted Saccade Amplitude (χ^2^(1) = 5.90, φ = 0.31, *p* = 0.0023) remained significant. The mixed model with adjusted Fixation Duration no longer showed a significant group difference (χ^2^(1) = 2.44, φ = 0.20, *p* = 0.12). However, the interaction between Task Scenario and Group was significant (χ^2^(12) = 25.45, φ = 0.64, *p* = 0.038), even after accounting for group differences in Total score.

#### The effect of saliency on task performance

 To evaluate the impact of saliency on participants’ performance we used a mixed model, which included Total Score as a target variable, Normalized scanpath saliency^44^ (NSS), calculated per task scenario, Task Scenario, and Group factors as fixed effects and Participant as a random effect (intercepts). The outcome of the model is presented in Table 3.

**Table 3:** Mixed Effect Model of NSS on Task score.

Dependent variable	Effect	χ^2^	Df	φ	p
Total Score *	Task Scenario	124.89	12	1.41	< 0.0001 ***
	NSS *	1.96	1	0.18	0.15
	Group	6.05	1	0.31	0.014 *
	NSS × Group	0.513	1	0.08	0.51

*Calculated in a Task Scenario.

The model suggests a difference in difficulty between task scenarios (the effect of Task Scenario on Total Score) and Total Score between the ADHD and control groups. However, the difference in Total Score was not associated with NSS, and there was no interaction between NSS and Group either.

NSS did not differ between the groups, neither across all 13 EPELI tasks*,* nor when distractor and non-distractor conditions were tested separately. It is noteworthy that on average NSS was close to zero in both groups (0.21 ± 1.4 in the ADHD group and 0.02 + 0.6 control group), which indicates that saliency had only a weak effect on gaze allocation overall.

#### The effects of object relevance

**Figure 4:**
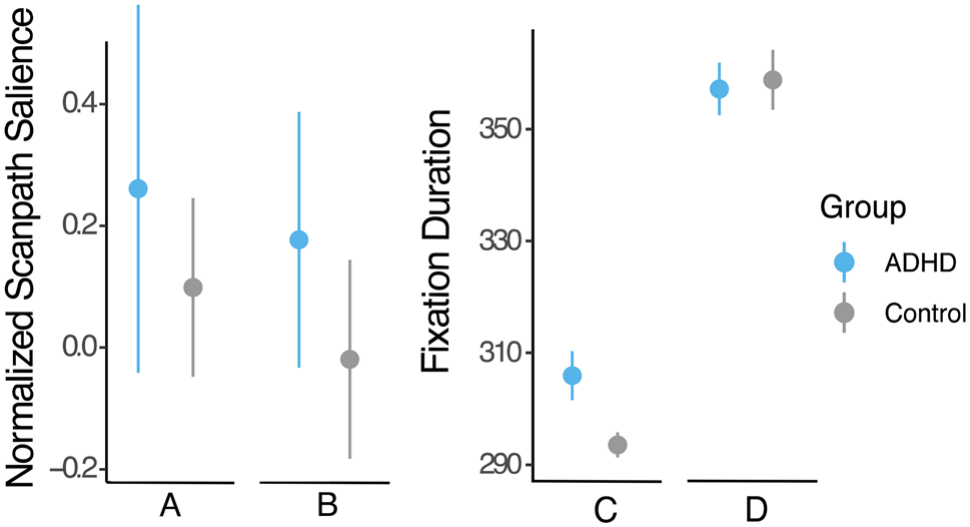
The left panel represents normalized scan path saliency averaged by Condition. (**A**) Task Scenarios without distractors, (**B**) Task Scenarios with distractors. The right panel shows fixation durations to different types of objects. (**C**) Task-irrelevant objects, (**D**) Task-relevant objects.

 To further investigate which factors drive the observed group differences in eye movements, we examined how the relevance of objects in the EPELI influenced attention allocation (see Fig. 4). Overall, Fixation Duration differed between task-relevant and task-irrelevant objects (Z = 8.6, *p* < 0.0001). However, no group differences were observed between the task-irrelevant and task-relevant objects. The clinical group had a tendency for longer Fixation Duration to the task-irrelevant objects as compared with the control group. However, this effect did not reach significant level (Z = 2.2, *p* = 0.060).

#### The effects of ambient versus focal processing

**Figure 5:**
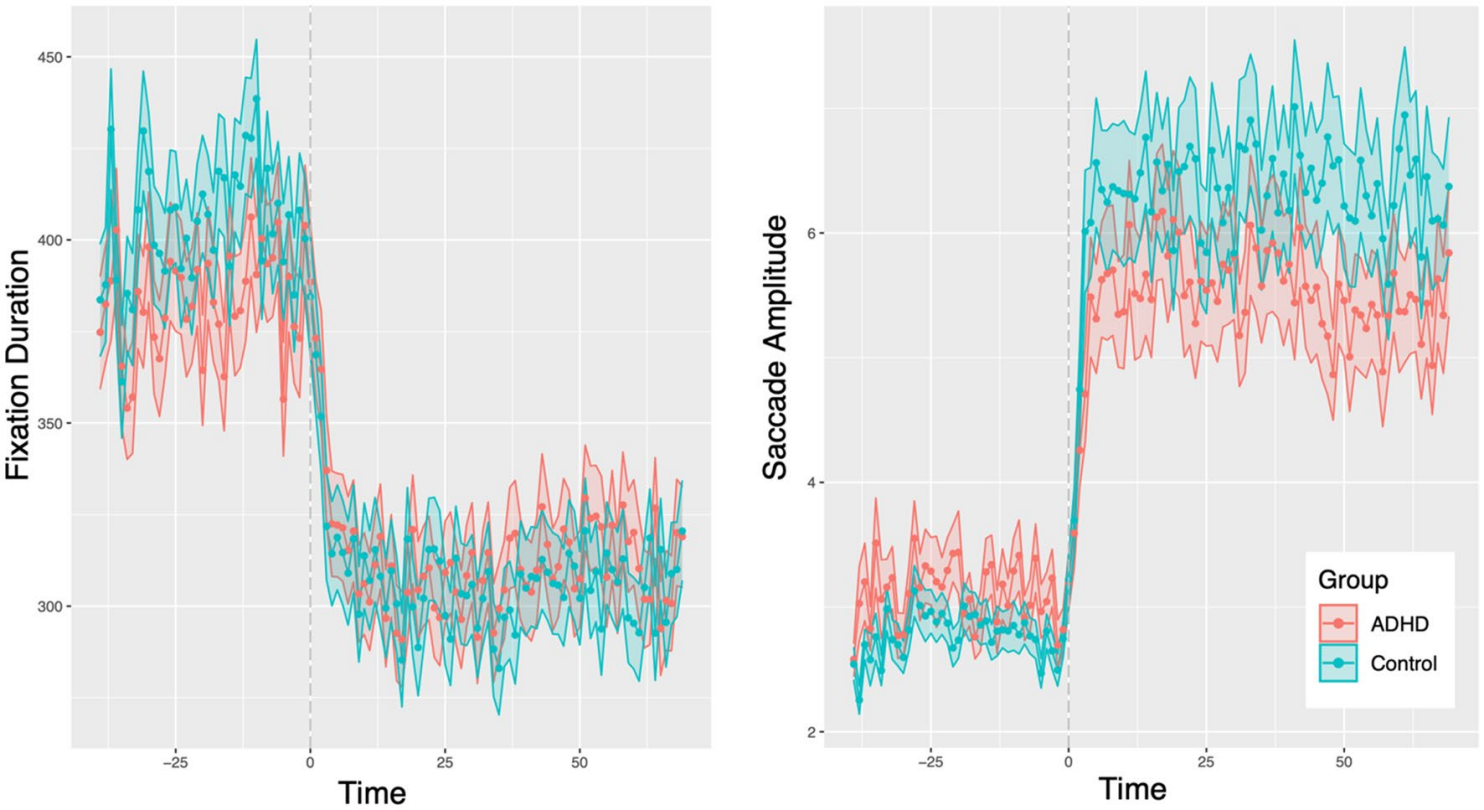
Eye movement characteristics over time. Zero time point indicates when instruction phase ended, and task execution phase started. (**A**) Fixation Duration, (**B**) Saccade Amplitude; for all features tested in the analysis see Fig. S1 in Supplementary Results: Ambient versus Focal Processing.

 Significant effect of time on eye movement properties was observed, suggesting that the participants were indeed switching between ambient and focal processing during task performance (see Fig. 5). However, no group differences in switches between the ambient and focal modes were detected. An additional analysis showed that the group differences in the eye movement features changed clearly when the participants proceeded from the instruction to task execution phase. The related results can be found in Supplementary Results section (Supplementary Results: Ambient versus Focal Processing).

### Shoot the Target task

Participants with ADHD had lower Total Score than participants in the control group (t(54) = 3.63, *p* = 0.0018). The group differences in the Head Rotation Angle and Head Rotation Speed were not statistically significant (see also Supplementary Results: Fig. S3).

A mixed linear model for the eye movement features showed that ADHD group had longer Fixation Duration (χ^2^(1) = 12.04, φ = 0.44, *p* = 0.0016), and shorter Saccade Duration (χ^2^(1) = 10.25, φ = 0.41, *p* = 0.0021) and Saccade Amplitude (χ^2^(1) = 4.17, φ = 0.26, *p* = 0.041) also in the Shoot the Target task.

Additional mixed model was conducted to accommodate for difference in data loss between the groups (see Methods: Processing of eye movement data). The model included percentage of invalid gaze samples as random effect, and showed that difference in Fixation Duration between the groups was not affected by the lost data (Group effect: χ^2^(1) = 8.43, *p* = 0.011, invalid data effect: χ^2^(1) = 0.02, *p* = 0.87). However, there was no significant Group effect in this model on Saccade Duration, (χ^2^(1) = 3.04, *p* = 0.12; invalid data effect: χ^2^(1) = 6.58, *p* = 0.03) nor on Saccade Amplitude (χ^2^(1) = 1.41, *p* = 0.23, invalid data effect was not significant either, χ^2^(1) = 1.40, *p* = 0.36).

The classification accuracy obtained with SVM 3 based on Shoot the Target eye movement data was 0.78 AUC, which was worse than SVM 2 (t(58) = 9.93, *p* < 0.0001) performance utilizing EPELI eye movement data.

### Discussion

Cognitive deficits and behavioral problems of children with ADHD are heterogeneous and idiosyncratic. Therefore, conventional experimental methods with restricted designs, constraining the heterogeneity and tapping only narrow symptom domains at a time, may not be optimally suited to support diagnostics^2,3^. The present study shows that recent advances in VR technology can be used to detect attentional and executive deficits in a complex ecologically valid setting, and with high precision. More specifically, we demonstrate several benefits of VR eye tracking in interactive and open-ended tasks mimicking real-world situations: (a) rich eye movement features during EPELI performance provided excellent classification accuracy (AUC), which was significantly higher as compared with task performance measures in a similar situation, (b) AUC was also significantly higher in a condition requiring active execution of goal-directed behavior (EPELI) than in a conventional visual search condition (Shoot the Target), (c) we tested a novel approach for analyzing eye movement behavior to pinpoint distinct goal-directed and stimulus-driven processes via task-relevance and saliency-based analyses in a naturalistic setting via EPELI task. This analysis suggested that aberrant eye movements observed in ADHD participants may not be restricted to any specific type of attention-related process that was examined, but rather rise from multiple factors dispersed over the whole task performance. Together these findings strongly advocate the benefits of VR eye tracking and naturalistic conditions in psychiatric research.

We used a recently developed VR paradigm, EPELI, which simulates everyday life situations. In this study, we first successfully replicated the behavioral results obtained in our earlier study^12^. All five performance measures showed group differences. Although task performance already successfully discriminated the two groups, utilizing eye movements still considerably improved the discriminative ability of the classifier, which reached 0.92 AUC with eye tracking measures only. Examination of the separate eye movement features revealed that the ADHD group demonstrated longer fixations and shorter saccades with lower amplitudes, as compared with the control group. Group differences in these parameters between ADHD children and controls have also been reported in prior eye tracking studies with restricted experimental designs not allowing active interaction with the environment^14,15^, although as our findings demonstrate, the manifestation of group differences in these parameters is dependent on the task context. Pointing the contextual effects is important as in majority of the tasks used for ADHD assessment, actions are restricted, interaction is limited, and required activity levels are low.

Longer fixations in the ADHD group across the whole experiment may reflect inefficient and superficial information processing. This interpretation is supported by computational modeling work on decision-making in ADHD^45,46^ and also consistent with the lack of group differences in fixations to relevant objects in this study (Fig. 5). That is, shorter fixations to target objects for ADHD children would have presumably indicated more superficial processing of target objects. As saccades are used to bring the gaze towards the objects that are relevant for our ongoing behavior^26^, their role in inefficient visual search is even more easily argued^47^. In concordance with our findings, also in other types of tasks fewer saccades within a task has been linked to successful task performance^48^. Overall, saccades becoming longer and fixations shorter can be used as an index for the development of visual attention^48,49^. Hence, our results might indicate that maturation of the visual system is delayed in ADHD children^48^.

The alterations in eye movements in ADHD were dispersed over the whole experiment rather than being associated with specific types of events or objects that were extracted to pinpoint goal-directed (e.g., relevant vs. irrelevant objects) and stimulus-driven (e.g., object salience related to perceptual features) processes underlying aberrant eye movements. We would like to note that execution of goal-directed behaviors is present throughout the EPELI task, also when the gaze is not in target objects. Hence, it is highly likely that the observed eye movement effects are related to goal-directed behavior, even though in this type of dynamic condition it appears to be the visual search rather than the depth of processing relevant objects that differentiate ADHD children from typically developing children. However, the lack of salience effect during active execution of goal-directed behaviors may indicate that such effects that are observed in conditions in which isolated and static stimuli are presented, do not appear in open-ended tasks where top-down processes could suppress salience effects^1^. Even though we observed trends pointing at possible small effects of bottom-up processes, we did expect larger role for stimulus-driven behaviors and therefore conclude that we did not find support for our original hypothesis here.

Another important limitation to consider is the moderate sampling rates of the available eye-tracking systems for VR headsets, including the one used in the present study. Low sampling rate does not allow to reliably detect shorter, more rapid saccades and is problematic for estimating peak velocity^50^, but it is suitable for evaluation of fixation duration, and properties of longer saccades, such as duration and amplitude. It was previously shown that even low-speed eye-trackers of 90–120 Hz allow to extract eye movement features with descent accuracy^51^.Despite some limitations, modern VR eye-trackers provide valuable opportunity to study natural behavior and obtain relevant information for further development of naturalistic methods. But as technology is constantly developing, replication with a higher sampling rate eye-tracker, when available, would be beneficial for more precise estimation of eye movement properties in naturalistic setting.

The group differences for three eye movement features had similarities between EPELI and Shoot the Target, even though these two tasks were quite different. This supports our interpretation above related to inefficient visual search. However, as we did not have a control condition where the role of attention would be separated from general eye movement programming and implementation, we cannot completely rule out the possible contribution of such lower-level processes on our findings^14^. Nevertheless, as classification of individual participants group status was clearly more reliable in EPELI where participants were allowed to navigate freely as compared to passive listening or simple visual search task, it is unlikely that problems in basic visuomotor processes would explain our findings to a considerable extent.

The effect of group on saccade duration and amplitude were significant even after correction for overall task performance, even though this made the effects somewhat smaller. In fixation duration, only interaction between task and group, not the main effect of group, showed a significant effect after regressing out the influence of task performance. This means in practice that the groups differed in fixation duration only in few task scenarios when the effect of task performance was accounted for. It is not surprising that eye movements are associated with task performance, as efficient visual attention plays a key role in EPELI task. Based on the present results it is difficult to fully understand whether the task performance is lower in ADHD participants because they have difficulties in visual attention that is observed in eye movements or whether their eye movements are different because they have difficulties in performing the task. What can be stated, however, is that the group differences in two out of three main eye movement features remain even after task performance is accounted for. Future studies separately manipulating the specific cognitive processes associated with task performance should be conducted to clarify the links between task performance and eye movement behavior. This could be achieved, for instance, by parametrically varying the memory demands (e.g., number of subtasks) vs. attentional demands (e.g., the amount of irrelevant visual information) in the EPELI game.

Even though the specific events did not seem to be driving the group differences in the present study, we successfully demonstrated that by utilizing VR, it is possible to connect eye movements to multiple features that are relevant in guiding human attention. This opens new means to approach aberrant goal-directed behavior in future clinical studies and could be useful in studies with other clinical groups too (e.g., autism, schizophrenia, mood disorders, neurodegenerative disorders).

Overall, we did observe switches between ambient and focal processing modes during task performance, and this was also affected by familiarity of the environment. However, even though extended performance variability was reported in a previous study with EPELI task^6^ and was also observed in conventional CPT task in the present dataset, we did not observe group differences eye movement fluctuations during the VR task. Future studies could look more closely into intra-individual variability metrics during performance of naturalistic VR tasks. However, it is possible that such effects are more prominent in conditions with stimulus poverty^23^.

It is possible that we did not detect some small effects (e.g., possible group differences in fixations to relevant vs. irrelevant stimuli) due to the relatively small sample size. Larger sample with more prominent symptom variability would have also been useful to be able to examine the role of various other factors possible influencing eye movement behavior in EPELI task (e.g., age, gender, cognitive abilities) as well as to pinpoint the links between specific symptoms and eye movement behaviors. It is also possible that some participants in our control group had significant psychiatric symptoms or other confounding medical issues, as we did not perform a comprehensive diagnostic interview for the control participants, but rather settled for standardized parent questionnaire (CBCL). A replication of the study with a larger sample and stricter control over control group comorbidities would be useful to confirm the obtained results and to see how well they generalize to a larger population.

## Conclusions

To conclude, our study showed that eye movements recorded in naturalistic setting provide a promising behavioral marker for ADHD assessment, which can be used to predict the diagnosis with excellent accuracy. Demonstrating the performance of VR eye tracking for differential diagnostics and reproducibility of the present results, however, needs further research. The aberrant characteristics of eye movements appear to be mostly related to inefficient visual search and highlighted when the task requires active execution of complex goal-directed behaviors. These promising findings strongly advocate the use of naturalistic VR tasks in future clinical research and development of diagnostic methods.

## Methods

### Participants

The participants were accepted to the control group if they (1) did not have any mental, behavioral, and neurodevelopmental disorders (F00–F99 in ICD-10), nor any diseases of the nervous system (diagnosis G00-G99), (2) they were not entitled to special support at school for other reasons. These criteria were controlled for via a parent questionnaire. Additional criteria for the ADHD group were: (1) ADHD diagnosis (F90) made by a licensed medical doctor and verified via the National Medical Database, (2) no diseases of the nervous system (diagnosis G00-G99 in ICD-10), nor any other mental, behavioral, or neurodevelopmental disorders (F00–F99) except ADHD, and two possible comorbid diagnoses, F93.89 (Emotional disorder with onset specific to childhood) and F93.9 (Unspecified childhood emotional disorder). These comorbidities were accepted for being commonly present in children with ADHD.

Five participants from the control group and one participant in the clinical group were excluded from the final sample for the following reasons. The control group: one participant from the control group was excluded because of difficulties with Finnish language due to significant time living abroad, and four were excluded due to technical problems. In the clinical group, one participant dropped out during the experiment and the related data was thus excluded. Possible comorbid disorders in the ADHD group were screened with MINI-KID Interview for Children and Adolescents 7.0—a structured diagnostic interview for ICD-10^52^. Based on the interview, four participants matched the criteria for oppositional defiant disorder (F91.3); three for unspecified conduct disorder (F 91.x); two for panic disorder (F41.0); two for unspecified affective disorders (F33.x); and one for chronic motor or vocal tic disorder (F95.1).

In the control group all participants were screened for possible ADHD symptoms with ADHD Rating Scale-IV (ADHD-RS; parent form)^53^. All but one participant scored below 85 percentiles in ADHD-R, which corresponds to low probability of having ADHD. Based on Child Behavior Checklist (CBCL)^54^, no participants in the control group had noticeable attention problems. However, two participants had clinically significant scores on Withdrawn/Depressed Scale two on Somatic Complains scale and one on Rule-Breaking and Aggressive Behavior Scale. None of them were excluded from the study.

In the clinical group, all children but three were taking medication to alleviate their ADHD symptoms, namely methylphenidate (29 children), amphetamine (four), Guanfacine (one). In addition, two participants with ADHD had risperidone prescription for behavioral problems and three had melatonin prescription for sleep problems. The participants were asked to take a break on their ADHD medication for 24 h before the measurement. In the control group, one participant was reported to use thyroxine, one was using budesonide against asthma symptoms, and one was taking an antihistamine drug.

Two participants had incomplete background data (one in the ADHD and one in the control group), but they were nonetheless included in the study.

Ethical approval for the study was received from the Ethics Committee of the Helsinki University Hospital (HUS/1589/2019). The research was performed in accordance with relevant guidelines and regulations, including the Declaration of Helsinki. Informed consent was obtained from all participants and their parents.

### EPELI task

EPELI is an open-ended VR task that was developed for the assessment of goal-directed behavior and related cognitive functions (e.g., attention, executive functions, and prospective memory) and validated in a previous study^12^. The game was implemented by Peili Vision Company (http://www.peilivision.fi/).

In EPELI, the child performs 13 different task scenarios that model everyday life chores. Each task scenario consists of an instruction phase, in which a dragon character describes the task scenario and its subtasks to the participant, and the execution phase, where the participant should perform the given subtasks. The game environment models a typical apartment with a child’s bedroom, living room, kitchen, parents’ bedroom, and a bathroom (for EPELI floor plan, see Seesjärvi and colleagues^12^). The participant does different subtasks by moving around the apartment and interacting with objects there. In total, the game includes 70 subtasks. Moreover, every other scenario includes extraneous visual and auditory distractors. Across all task scenarios, 47 objects were relevant (necessary for completing the tasks), and 243 were irrelevant regarding task performance. A detailed description of the game can be found in Seesjärvi and colleagues^12^, along with Supplementary Methods: EPELI section.

The five EPELI measures established by Seesjärvi and colleagues^12^ included: Total score (percentage of subtasks completed successfully during the game), Task efficacy (percentage of relevant actions, i.e. the actions that were necessary to perform to complete the subtask out of all actions excluding clicks on teleport waypoints), Navigation efficacy (total score divided by distance covered by moving around the apartment and distance to an object at the moment of interaction), Controller Motion (sum of controller angular movements), and Total Actions (total number of controller clicks during task instruction and execution). Additionally, Total score was calculated on each task scenario separately for Saliency Analysis (see Supplementary Methods: Normalized Scanpath Saliency).

The EPELI game was followed by related assessment containing, for instance, rating of the object familiarity, instruction recall task (see Supplementary Methods: EPELI).

### Shoot the Target task

Shoot the Target is a VR visual search task, implemented by Peili Vision Company^55^. In this task, the participants were instructed to search for target objects appearing in the surrounding visual space and shoot those by orienting their gaze to the object (see Supplementary Methods: Shoot the Target for details of the task and https://www.youtube.com/watch?v=zbgZuklqRac for a video illustration).

The behavioral measures analyzed in the task included: Total Score (correctly selected targets divided by the number of presented targets plus false alarms), Head Rotation Angle (sum of head rotation angle during the game), and Head Rotation Speed (average of head angular speed).

### Equipment

To conduct the VR tasks, we used Pico Neo 2 Eye HDM with a resolution of 1920 × 2160 pixels per eye. The refresh rate of the device is 75 Hz and field of view 101°. Gaze position was recorded with Tobii 90 Hz eye tracker with 0·5° stated system accuracy integrated into the VR headset. The tasks were launched and observed by the experimenter via Samsung Galaxy Tab S3 tablet.

### Procedure and other data that was collected

The session included also Similarities and Matrix reasoning subtests of the Finnish version of WISC-IV^41^. In addition, one parent or caregiver was asked to fill in a set of questionnaires: ADHD Rating Scale-IV (ADHD-RS)^53^, the Behavior Rating Inventory for Executive Functions (BRIEF)^56^, the Child Behavior Checklist (CBCL)^54^, and the Executive Questionnaire of Everyday LIfe (EQELI)^12^.

### Processing of eye movement data

Five-point eye-tracking calibration was performed before each VR task with Tobii inbuild software. Among the participants with eye-tracking data, two were excluded from the EPELI eye movement analysis based on high rate of invalid samples (80 and 30 precents). There were no differences in the percentage of the lost samples between the groups after the exclusion, and overall percentage of lost data was low (75 percentile – 5%, maximum – 16%). In the Shoot the Target data, the overall proportion of invalid gaze sample was rather small in both groups (75 percentile – 1%, the maximum data loss – 10%). However, the ADHD group had a higher percent of lost data points (Z = 3, *p* = 0.002), so in the result section, we report the outcome of mixed model including percentage of invalid gaze samples as a random effect.

Saccades and fixations were detected from raw gaze data with a modification of Engbert and Kliegl’s algorithm^57^ for detecting eye movement events with unconstrained head movements^58^. Head compensation was conducted by mapping raw gaze positions to world space coordinates. The eye movement features included were fixation duration, saccade duration, and saccade amplitude. Peak velocity was excluded from the analysis due to low sampling rate of the eye-tracker. Smooth pursuit detection was not included due to the lack of objects moving with suitable speed for smooth pursuit in the game.

Saccades with duration below 22 ms (the threshold was selected as 1/sampling rate * 2), and with amplitude below 0.75 degree (following the threshold suggested by Larsson and colleagues^58^) were excluded from the analysis. In addition to that, saccades over 300 ms were considered as noise and therefore excluded, and the maximum acceleration accepted for a saccade was 36,000 degree/sec^2^^58^. Similar cleaning procedure was applied to the fixations (duration within 100–1000 ms range was accepted.

Only eye movements during execution phase of a Task Scenario were included in the EPELI eye movement analysis if not otherwise was specified. Fixations on teleport waypoints, walls and floor were not considered as objects and respectively were not included in the analysis related to relevance of the objects. They were also excluded in calculation of saccade proportion within the same object (for ambient versus focal analysis). However, a separate analysis was performed for non-object related eye movements, which included fixations on the floor and walls. This was included to clarify the role of object-interaction in group differences (see Supplementary Results: Eye movement effects outside objects).

### Saliency analysis

Participant’s gameplay was broadcasted to a tablet and recorded as a video. The video was cut into fragments corresponding to each task automatically based on time stamps. Each video was processed with MT_TOOLS toolbox^59^ for creating saliency maps based on color, orientation, intensity, flickering, and motion (https://www.brainreality.eu/mt_tools/). Normalized Scanpath Saliency (NSS) was used as a saliency index^44^ (also see Supplementary Methods: Normalized Scanpath Saliency).

### Analysis of ambient versus focal mode

For this analysis, the time a participant was performing a subtask was divided into 1-s bins. For each bin, mean Fixation Duration, Number of Fixations, mean Saccade Amplitude and proportion of Saccades within the Same Object were calculated as the features reflecting processing mode. The group differences in switches between the modes were tested by effect of interaction between Group and Time on the selected features (see Supplementary Methods: Analysis of Ambient vs Focal Processing).

### Statistical testing

 Supplementary methods Statistical Analysis contains a detailed description of statistical tests and models. All Reported *p* values were adjusted for multiple comparisons with False Discovery Rate correction^60^.

### Classification

An SVM classifier^42^ was trained in one cross-validation loop with automatic hyper-parameter search and evaluated in a separate loop with tenfold cross-validation^61^. The training and evaluation were implemented the Python package sklearn version 1.0.2^42^. The data was first separated in ten cross-validation samples, then the data was scaled, decomposed with principal component analysis (PCA)^62^, and used as input for an SVM classifier. Scaling and PCA were fit based on training data only. Number of PCA components, SVM kernel, C, and gamma parameters were selected automatically with grid search. Statistical differences in classifiers’ performance were tested on 30 iteration Bootstrap Cross-Validation^63^, tenfold with two-sided *t*-test.

As performance evaluation, averaged AUC score^64^ on validation sample is reported for each classifier. To calculate this score, a ROC curve is first created by plotting true positive rate (sensitivity) against false positive rate (one minus specificity) at various decision thresholds. Total area under the curve (AUC) ranging from 0.5 (random classification) to 1.0 (perfect classification) is used for evaluating classifier performance^64^.

## Supplementary Information

Supplementary Information.

## Data Availability

The data in anonymized form is available for research purposes upon reasonable written request to the corresponding author.

## Code availability

The code used to perform the analyses and generate the plots is available by request to the corresponding author.
