# Supplementary material for: Eye movement behavior in a real-world virtual reality task reveals ADHD in children
Source: Sci Rep. 2022 Nov 24;12:20308. doi: 10.1038/s41598-022-24552-4 (PMC9700686; doi:10.1038/s41598-022-24552-4)
Supplement: Supplementary file 1 — Supplementary Information. [file 41598_2022_24552_MOESM1_ESM.docx]

**“Eye movement behavior in a real-world virtual reality task reveals ADHD in children"**

**Supplementary Materials**

**Table of Contents**

[SUPPLEMENTARY METHODS 2](#_Toc116228023)

[EPELI 2](#_Toc116228024)

[Task description 2](#_Toc116228025)

[Additional distractors 2](#_Toc116228026)

[Main measures 2](#_Toc116228027)

[After-game assessment 3](#_Toc116228028)

[Shoot the Target 3](#_Toc116228029)

[Task description 3](#_Toc116228030)

[Game parameters 3](#_Toc116228031)

[Instruction for the game 4](#_Toc116228032)

[Normalized Scanpath Saliency 4](#_Toc116228033)

[Analysis of Ambient vs Focal Processing 4](#_Toc116228034)

[Statistical Analysis 5](#_Toc116228035)

[Implementation 5](#_Toc116228036)

[Summary of the statistical tests included in the study 5](#_Toc116228037)

[Classification 6](#_Toc116228038)

[SUPPLEMENTARY RESULTS 7](#_Toc116228039)

[Questionnaires 7](#_Toc116228040)

[Statistical Analysis Adjusted for Gender 7](#_Toc116228041)

[EPELI results 7](#_Toc116228042)

[Shoot the Target results 8](#_Toc116228043)

[Correlation between Eye Movements and ADHD-RS score 8](#_Toc116228044)

[Distractor Effect 8](#_Toc116228045)

[Eye movement effects outside objects 9](#_Toc116228046)

[Ambient versus Focal Processing 9](#_Toc116228047)

[The effects of Instruction versus Execution phase 11](#_Toc116228048)

[Shoot the Target 12](#_Toc116228049)

[References 13](#_Toc116228050)

# SUPPLEMENTARY METHODS

## EPELI

### Task description

The child version of EPELI includes 13 task scenarios and a practice scenario at the beginning of the game. During the practice scenario, the participant learned how to navigate the environment, manipulate the objects, and use the watch to monitor the time. A cartoon dragon character guided the player during the practice.

Each task scenario had a general topic (e.g., Morning Routines or Coming Back From School), and consisted of four to six subtasks (e.g., put backpack to our room, wash your hands, eat lunch). The scenarios had two phases: instruction phase, and execution phase. During the instruction phase, the dragon described the topic of task scenario and its subtasks to the participant; teleportation and interaction with the objects were disabled during this phase. Instruction phase followed with the execution phase, where the participant had to perform the given subtasks. It ended if the participant performed all the subtasks correctly, or if the time limit of 90 seconds was reached. The total duration of the game was 25–35 minutes, depending on participant’s performance.

The order of the task scenarios was counterbalanced in a way that half of the participants performed the tasks in the reversed order.

### Additional distractors

The visual distractors included several additional objects haphazardly located all over the flat, and the auditory distractors consisted of ambient sounds (such as car horn honking or ambulance siren coming outside the apartment), while one distractor was audio-visual (a buzzing fly). Every other task scenario had additional auditory and visual distractors. Due to the counterbalancing, half of the participants performed specific task scenarios with the additional distractors, while the other half had distractors in the rest of the scenarios.

### Main measures

In order to replicate the results concerning the behavioral differences found in the previous study, we used the main five EPELI measures established by Seesjärvi and colleagues (1). The measures included: Total score (percentage of subtasks completed successfully during the game), Task efficacy (percentage of relevant actions, i.e. the actions that were necessary to perform to complete the subtask out of all actions excluding clicks on teleport waypoints), Navigation efficacy (total score divided by distance covered by moving around the apartment and distance to an object at the moment of interaction), Controller Motion (sum of controller angular movements), and Total Actions (total number of controller clicks during task instruction and execution). Additionally, Total score was calculated on each task scenario separately for Saliency Analysis (see below).

### After-game assessment

EPELI game was followed by a related assessment. In the Object Recognition and Rating Task the participants were presented with a small subsample of objects (including relevant and irrelevant ones) from the game and asked to name them and evaluate how fun the object was with 5-point Likert scale. In addition to that, after EPELI session participants performed a Repetition task (repetition of sentences resembling the instruction given by the dragon character in EPELI), see (1) for details. The assessment also included a shortened version of the Presence Questionnaire 3.0 (2), the Simulator Sickness Questionnaire (3), and a gaming experience questionnaire (see (1), Supplementary Table 5). Total duration of the after-game assessment was 10–15 minutes. More details regarding the after-game assessment will be published in a separate study (see preregistration https://osf.io/vzxsq/?view_only=c8a70ca2b21c410dbbb18ce3b024bb31).

## Shoot the Target

### Task description

During the task, the target type alternated among six different shapes (target switch was associated with an auditory cue). Thus, an object of a particular shape at the time was a target, while objects with other shapes were non-targets. The information regarding target type was constantly available below the actual stimulus area. All objects had one of the six different colors assigned randomly, but the color was not a target feature in the visual search. The objects that had been selected broke into pieces and disappeared. If an object was not shot, it disappeared after three seconds. A new object appeared in a random spatial location every half a second until the ten objects limit was reached.

### Game parameters

Number of presented stimuli 400

Number of targets 100

Number of stimulus shapes 6

Number of stimulus colors 6

Stimulus lifetime 3 s

Time between stimuli 0.5 s

Maximum simultaneous stimuli 10

Color assigned to a stimulus at random

Spawning pause after target shape change 2 s

### Instruction for the game

The instruction was read to the participant by the experimenter. The English translation:

“In this game, you need to destroy asteroids to protect Earth. You have a powerful laser gun, that can blow up an asteroid. To activate the gun: select an asteroid with your gaze and look at it for 2 seconds. Be careful. To finish your mission successfully, you need to destroy asteroids only of a specific shape. If you look down, you see the screen, that shows you, which shape you should target. Remember, asteroids can appear also outside your visual field in the front space, also in the far left or right, turn your head to see more. If you hear a sound, it's now the time to blow new kids of asteroids! Check the target shape again, it has changed. Don't hesitate to ask questions if something is unclear. You'll have a chance to practice before we start.”

## Normalized Scanpath Saliency

In order to calculate Normalized Scanpath Saliency (NSS) each saliency map was normalized to have zero mean and standard deviation one (4). Normalized saliency values were extracted at each location that corresponded to a participant's fixation. Mean of these values per each task scenario were taken as NSS measure. In this measure, values greater than zero correspond to a visual scanpath guided by saliency, value of zero indicate absence of connection between saliency and the scanpath, and NSS below zero corresponds to active avoidance of salient locations (4).

## Analysis of Ambient vs Focal Processing

For the analysis of ambient *vs.* focal processing, the time during which a participant was performing a subtask was divided into bins of 1 sec duration. Only complete bins were included in the analysis (so, if the subtask took 1.23 sec to complete, the first second is used for bin 1, and the rest 0.23 sec was removed). As the number of observations decayed exponentially over time, only the bins that included number of observations within 1.5 interquartile range (IQR) were included in the analysis (which resulted in the first 25 seconds). If a fixation or a saccade overlapped with two bins, it was included to the earlier bin.

Eye movements related to the floor and walls were excluded from the analysis. Proportion of within object saccades was calculated as the number of consecutive fixations on the same object divided by the number of consecutive fixations on different objects. The object label was mapped to gaze ray by Unity.

Four mixed models were used to test group difference in switches between ambient and focal processing in reaction to game events and distractors over time at the level of single EPELI subtasks. Each model included one of the following dependent variables:

Number of Fixations

Saccade Amplitude

Fixations Duration

Proportion of Within Object to Between Object Saccades

Each model also includes the following Fixed Effects:

Number of Teleportations

Number of Actions

Condition (distractor versus non-distractor Task Scenario)

EPELI Time (time from the start of EPELI)

Subtask Time (time from the last completed subtask)

Group

Participant and Task Scenario were included as Random Effects.

## Statistical Analysis

### Implementation

Statistical analyses were performed with R version 4.2.0. Group differences were tested with t-test (for normally distributed variables; R function t.test), and with Mann–Whitney U test (for non-normally distributed data; function wilcoxonZ from package “rcompanion”, z-score statistic is reported). Mixed models used in the study were linear mixed effects models, which were fitted with packages lme4, and tested with Anova function in car package. Reported p values were adjusted for multiple comparisons with False Discovery Rate correction if not otherwise specified (with R function p.adjust from package “stats").

### Summary of the statistical tests included in the study

Fisher’s Exact Test was used to test statistical difference in Gender balance between the Groups.

A summary of the difference in means tests used in the study is provided in Table S1. T-tests/Mann–Whitney U tests were used to test the difference between Groups in the following Dependent Variables (see Materials and Methods section in the main text for abbreviation expansion and detail):

1) Background variables (viz. Age, Parental Income, WISC-IV Matrix Reasoning, WISC-IV Similarities, ADHD-RS, CBCL, BRIEF, EQELI)

2) Five Main EPELI Measures (viz. Total Score, Total Efficacy, Navigation Efficacy, Controller Motion, and Total Actions)^1^

3) Three Eye Movement Measures (viz. Fixation Duration, Saccade Duration, and Saccade Amplitude) for each group of EPELI objects separately (attractive and relevant; attractive and irrelevant; non-attractive and relevant; non-attractive and irrelevant objects)

4) Three Eye Movement Measures for two EPELI Conditions (distractor vs non-distractor)

5) Normalized Scanpath Saliency

6) Shoot The Target Behavioral Measures (viz. Total Score, Head Rotation and Head Rotation Speed)^1^

^1^ additional analysis adjusted for Gender is provided for these variables in Supplementary Results

**Table S1**. Summary of the mixed models tested in the study.

| **Dependent Variable** | **Fixed Effects** | **Random Effects** |
| --- | --- | --- |
| Three Eye Movement Measures (EPELI) ^1,2^ | Task Scenario, Group, Task Scenario × Group | Participant, Task Scenario Order |
| Three Eye Movement Measures (EPELI) | ADHD-RS total score | Participant, Task Scenario, Task Scenario Order |
| Three Eye Movement Measures (Shoot the Target) ^1^ | Group | Participant |
| Three Eye Movement Measures (Shoot the Target) | Group | Participant, Percentage of Invalid Gaze Samples |
| Three Eye Movement Measures (EPELI) on non-object spaces | Group | Participant, Task Scenario |
| Total Score (in a Task Scenario) | Task Scenario, NSS, Group, NSS × Group | Participant |
| Three Eye Movement Measures (EPELI) | Phase (instruction vs execution), Group, Phase × Group | Participant, Task Scenario, Task Scenario Order |
| Ambient versus focal analysis: | | |
| Number of Fixations, Saccade Amplitude, Fixations Duration, Proportion of Within Object to Between Object Saccades – all within 1 s bin | N of Teleportations, N of Actions, Distractor Condition, EPELI Time, Subtask Time, Group, Subtask Time × Group | Participant, Task Scenario |

^1^ additional analyses adjusted for Gender is provided for these variables in Supplementary Results

^2^ additional analyses adjusted for performance (Total Score) is also provided in the manuscript

## Classification

In total, four Support Vector Machine (SVM) (5) classifiers were tested in the study. Participants without eye tracking data were excluded from the classification analysis, so that comparisons between SVM results were not influenced by difference in the datasets. SVM 1 was trained on the five main EPELI performance measures (Total Score, Task Efficacy, Navigation Efficacy, Controller Movement, and Total Actions). SVM 2 classifier was trained with the same procedure on three eye movement metrics (Fixation Duration, Saccade Duration and Saccade Amplitude) averaged on each EPELI task scenario. Additional SVM was trained on the eye movements during instruction phase of a task scenario (see Supplementary Results). SVM 3 was trained on the same eye movement metrics obtained from data of Shoot the Target task.

# SUPPLEMENTARY RESULTS

## Questionnaires

As expected, the ADHD participants had higher score on the ADHD-RS questionnaire completed by parents (t(66)=14.63, p<0.0001), as well as higher level of psychiatric symptoms reported in the CBCL questionnaire (t(59)=8.85, p<0.0001), and executive function problems reported with BRIEF (t(69)=12.93, p<0.0001) and EQELI questionnaires (t(68)=9.35, p<0.0001).

The participants reported only negligible symptoms in the Simulator Sickness Questionnaire (average scores 0.65 (1.0) and 0.67 (1.1) in the ADHD and control group, while the maximum possible score is 14). The level of immersion in EPELI was evaluated high in the Presence Questionnaire (with average scores 70.4 (15.6) and 72.2 (10.5) respectively, out of maximum 84). All objects were well recognized by the participants, and familiarity of the tasks did not differ between the groups (see also Table 1 in the main text).

## Statistical Analysis Adjusted for Gender

### EPELI results

In addition to the main analysis, we tested difference in the five Main EPELI Measures adjusted by gender imbalance between the groups. It was tested with a mixed model which included Group as a fixed effect and Gender as random effect. The differences in Total Score (χ²(1)=7.06, φ=0.31, p=0.0098) Task Efficacy (χ²(1)=9.46, φ=0.36, p=0.0067), Navigation Efficacy (χ²(1)=7.90, φ=0.33, p=0.0082), and Total Actions (χ²(1)=9.00, φ=0.35, p=0.0067) remained significant, but not the difference in Controller Motion (χ²(1)=3.46, φ=0.22, p=0.063).

The models testing the difference between EPELI Eye Movement Measures adjusted by Gender mainly replicated the results reported in the main text of the paper: There was a significant Group effect on Saccade Duration (Group: χ²(1)=14.75, φ=0.48, p=0.00037, Group × Task Scenario p=0.50), and Saccade Amplitude (Group: χ²(1)=5.67, φ=0.30, p=0.017, Group × Task Scenario χ²(12)=22.61, φ=0.60, p=0.047). Group effect on Fixation Duration did not reach significance, however there was a significant interaction effect between Group and Task Scenario (Group: χ²(1)=3.78, φ=0.24, p=0.052, Group × Task Scenario χ²(12)=29.45, φ=0.68, p=0.010).

### Shoot the Target results

The models testing the difference between the Eye Movement Measures adjusted by Gender showed significant Group effect on Fixation Duration (χ²(1)=10.978, φ=0.42 , p=0.0028), Saccade Duration (χ²(1)=8.21, φ=0.36, p=0.0063), but not Saccade Amplitude (χ²(1)=2.98, φ=0.22, p=0.085). Among Shoot the Target performance measures, only Total Score was significant after adjustment by Gender (χ²(1)=14.72, φ=0.49, p=0.00038).

## Correlation between Eye Movements and ADHD-RS score

A mixed model was used to test the association between participant’s ADHD-RS and the eye movement features. The model showed that ADHD-RS was a significant predictor of Saccade Duration (χ²(1)=22,70, φ=0.60, p<0.0001), and Saccade Amplitude (χ²(1)=8.39, φ=0.36, p=0.0038), but not for Fixation Duration (χ²(1)=2.23, φ=0.19, p=0.14). However, interpretation of this result is limited, due to a floor effect in ADHD-RS score in the control group (Figure S1).

**Figure S1.** Distribution of ADHD-RS raw total score in the study sample.

## Distractor Effect

The effect of distractors was tested with mixed models that included Condition (distractor vs. non-distractor), and Group as fixed effects, and Participant as a random effect. The main effect of Condition was observed for Fixation Duration (χ²(1)=23.92, φ=0.62, p<0.001), but not for the other eye movement features. There was no Group × Condition interaction for any of the eye movement features.

## Eye movement effects outside objects

An additional mixed model testing whether similar effects in eye movements were observed also when gaze was directed to floor or walls had either Fixation Duration, Saccade Duration or Saccade Amplitude as a dependent variable, Group as fixed effect, and Task Scenario and Participant as random effects. The model indicated that the participants with ADHD have longer Fixation Duration (χ²(1)=5.29, φ=0.29, p=0.032) and shorter Saccade Duration (χ²(1)=10.8, φ=0.41, p=0.0030) than those in the control group. The difference in Saccade Amplitude was not significant after correction for multiple comparisons (φ=0.15, p=0.23).

## Ambient versus Focal Processing

The outcome of the mixed model analysis of switches between ambient and focal processing is presented in Table S2 below and in Figure S2. The result showed that there were changes in the processing mode over time (Subtask Time for all the eye movement features except Saccade Amplitude, and EPELI Time for Saccade Amplitude had significant effect on the eye movements). The results also indicated significant effect of events (Number of Teleports, Number of Actions, and Distractor Condition) on eye movement properties related to the two processing mods. But the model outcome did not indicate the differences in the temporal dynamic between the groups (no significant Subtask Time × Group interactions). However, we observed and strong effect on the eye movement related to the difference between instruction and task execution phase (see below).

**Table S2.** Summary of ambient vs focal processing modelling

| **Dependent Variable** | **Effect** | **χ²** | **df** | **p^1^** |
| --- | --- | --- | --- | --- |
| Fixations Duration | N Teleportations | 0.09 | 1 | 0.76 |
|  | N Actions | 57.44 | 1 | <0.0001*** |
|  | Distractor Condition | 5.05 | 1 | 0.025* |
|  | EPELI Time | 3.69 | 1 | 0.055 |
|  | Subtask Time | 19.91 | 1 | <0.0001**** |
|  | Group | 0.14 | 1 | 0.71 |
|  | Subtask Time × Group | 0.33 | 1 | 0.57 |
| Number of Fixations | N Teleportations | 85.71 | 1 | <0.0001*** |
|  | N Actions | 12.40 | 1 | 0.00043*** |
|  | Distractor Condition | 16.01 | 1 | <0.0001*** |
|  | EPELI Time | 3.03 | 1 | 0.082 |
|  | Subtask Time | 5.29 | 1 | 0.021* |
|  | Group | 0.15 | 1 | 0.70 |
|  | Subtask Time × Group | 0.76 | 1 | 0.38 |
| Saccade Amplitude | N Teleportations | 7.39 | 1 | 0.0066** |
|  | N Actions | 8.68 | 1 | 0.0032** |
|  | Distractor Condition | 0.16 | 1 | 0.69 |
|  | EPELI Time | 7.64 | 1 | 0.0057** |
|  | Subtask Time | 1.38 | 1 | 0.24 |
|  | Group | 6.68 | 1 | 0.0097** |
|  | Subtask Time × Group | 2.08 | 1 | 0.15 |
| Proportion of Saccades within the Same Object | N Teleportations | 408.15 | 1 | <0.0001*** |
|  | N Actions | 86.99 | 1 | <0.0001*** |
|  | Distractor Condition | 55.98 | 1 | <0.0001*** |
|  | EPELI Time | 40.91 | 1 | <0.0001*** |
|  | Subtask Time | 78.09 | 1 | <0.0001*** |
|  | Group | 1.08 | 1 | 0.30 |
|  | Subtask Time × Group | 0.009 | 1 | 0.92 |

**^1^** p-values reported in the table were not adjusted for multiple comparisons.


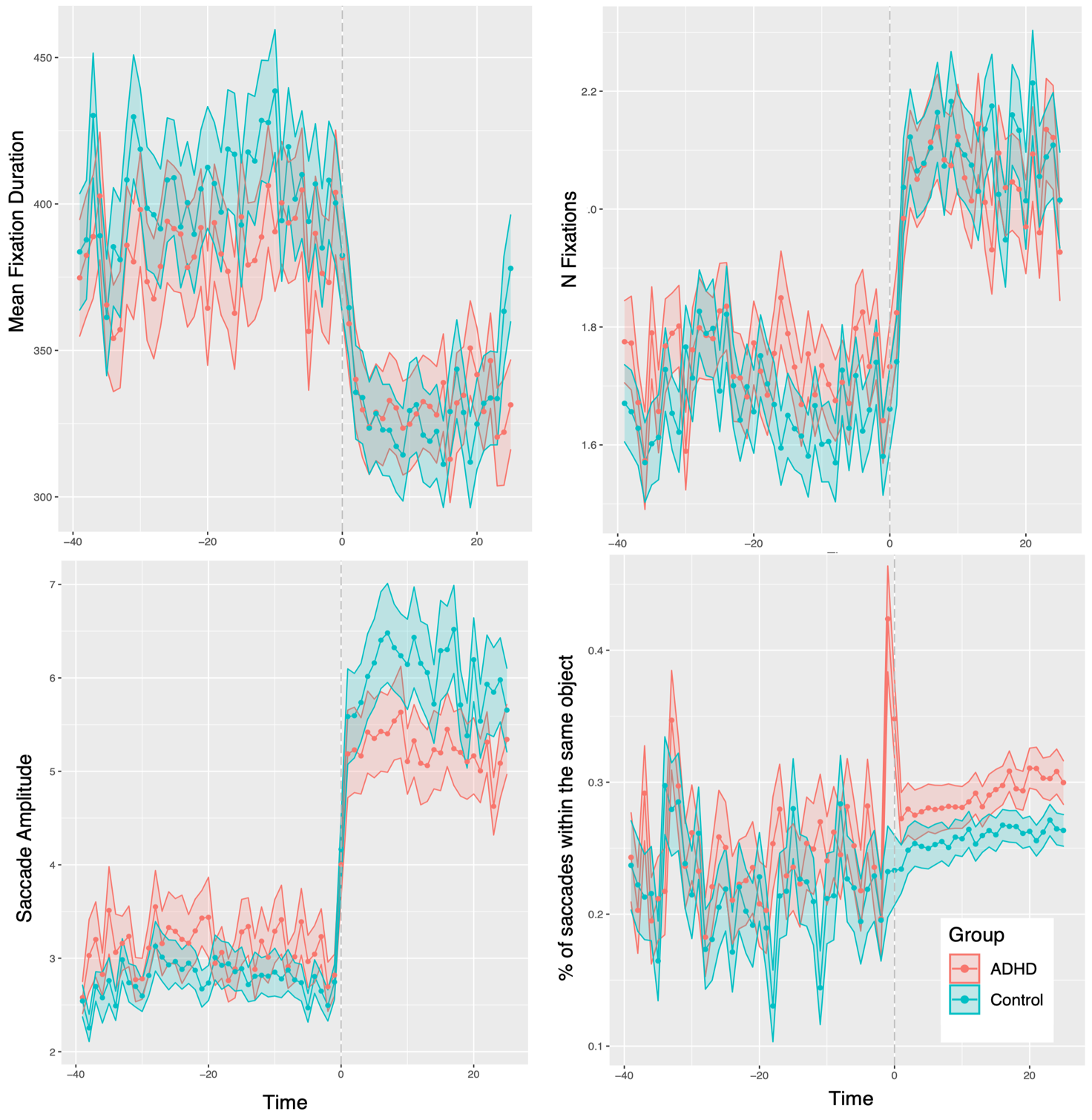


**Figure S2.** Change of the eye movement parameters over time while participants performed a subtask (time from 0 onwards). The figure also shows the data during the Instruction Phase (time below 0). The variance is expressed as the standard error of mean. A - Fixation Duration, B - Number of Fixations, C - proportion of saccades within the same object to between objects saccades, D - Saccade Amplitude.

## The effects of Instruction versus Execution phase

To test the influence of a Task Scenario Phase on the eye movement behavior and its interaction with Group factor, we conducted a mixed model analysis that included Phase and Group as fixed effects and Participant, Task Scenario, and Task Order as random effects. The models revealed a large effect of Phase on all three eye movement features, and a significant interaction between Phase and Group (see Table S3).

**Table S3.** Mixed models of Task Scenario Phase effects on the eye movements.

| **Dependent Variable** | **Effect** | **χ²** | **Df** | **φ** | **p** |
| --- | --- | --- | --- | --- | --- |
| Fixation Duration | Phase | 2877 | 1 | 6.76 | <0.0001*** |
|  | Group | 0.4 | 1 | 0.08 | 0.52 |
|  | Phase × Group | 57.7 | 1 | 1.11 | <0.0001*** |
| Saccade Duration | Phase | 1156 | 1 | 4.28 | <0.0001*** |
|  | Group | 15.4 | 1 | 0.49 | 0.00025** |
|  | Phase × Group | 30.3 | 1 | 0.69 | <0.0001*** |
| Saccade Amplitude | Phase | 4090 | 1 | 8.06 | <0.0001*** |
|  | Group | 6.5 | 1 | 0.32 | 0.017** |
|  | Phase × Group | 128.6 | 1 | 1.43 | <0.0001*** |

An additional SVM trained on eye movements during Instruction Phase resulted in Area Under Curve (AUC) score 0.79 that was lower than AUC for SVM 2 (t(58)=7.79, p<0.0001)

## Shoot the Target

**The figure illustrates group differences in the main behavioral variables of the Shoot the Target task. Among these three variables, only the difference in Total Score was significantly different between the groups (t(54)=3.63, p=0.0018).

**Figure S3.** Total Score, Head Rotation, and Head Rotation Speed in the Shoot the Target task.

# References

1. Seesjärvi, E., Puhakka, J., Aronen, E. T., Lipsanen, J., Mannerkoski, M., Hering, A., ... & Salmi, J. (2021). Quantifying ADHD symptoms in open-ended everyday life contexts with a new virtual reality task. *Journal of Attention Disorders*, 10870547211044214.

2. Witmer, B. G., Jerome, C. J., & Singer, M. J. (2005). The factor structure of the presence questionnaire. Presence: Teleoperators and Virtual Environments, 14(3), 298–312. <https://doi.org/10.1162/105474605323384654>

3. Kennedy, R. S., Lane, N. E., Berbaum, K. S., & Lilienthal, M. G. (1993). Simulator sickness questionnaire: An enhanced method for quantifying simulator sickness. *International Journal of Aviation Psychology,* 3(3), 203–220. https://doi. org/10.1207/s15327108ijap0303

4. Peters, R. J., Iyer, A., Itti, L., & Koch, C. (2005). Components of bottom-up gaze allocation in natural images. *Vision research*, **45**(18), 2397-2416.

5. Pedregosa, F., Varoquaux, G., Gramfort, A., Michel, V., Thirion, B., Grisel, O., ... & Duchesnay, E. (2011). Scikit-learn: Machine learning in Python. *The Journal of Machine Learning Research*, **12**, 2825–2830.
